# A hydroponics based high throughput screening system for Phytophthora root rot resistance in chickpea (*Cicer arietinum* L.)

**DOI:** 10.1186/s13007-019-0463-3

**Published:** 2019-07-26

**Authors:** Amritha Amalraj, Julian Taylor, Tim Sutton

**Affiliations:** 1 School of Agriculture Food and Wine, University of Adelaide, Waite Campus, PMB1, Glen Osmond, SA 5064 Australia; 2 South Australian Research and Development Institute, GPO Box 397, Adelaide, SA 5001 Australia

**Keywords:** PRR resistance in chickpea, *P. medicaginis* zoospores production, PRR phenotyping method, Hydroponics screening system, Plant survival traits, Kaplan–Meier (KM) estimates of survival probability, Linear mixed model, Whole genome QTL analysis, Combined hydroponics and field trait model

## Abstract

**Background:**

Phytophthora root rot (PRR) caused by *P. medicaginis* is a major soil borne disease in chickpea growing regions of Australia. Sources of resistance have been identified in both cultivated and wild *Cicer* species. However, the molecular basis underlying PRR resistance is not known. Current phenotyping methods rely on mycelium slurry or oospore inoculum. Sensitive and reliable methods are desirable to study variation for PRR resistance in chickpea and allow for a controlled inoculation process to better capture early defence responses following PRR infection.

**Results:**

In this study, a procedure for *P. medicaginis* zoospore production was standardized and used as the inoculum to develop a hydroponics based *in planta* infection method to screen chickpea genotypes with established levels of PRR resistance. The efficiency of the system was both qualitatively validated based on observation of characteristic PRR symptom development, and quantitatively validated based on the amount of pathogen DNA in roots. This system was scaled up to screen two biparental mapping populations previously developed for PRR studies. For each of the screenings, plant survival time was measured after inoculation and used to derive Kaplan–Meier estimates of plant survival (KME-survival). KME-survival and canker length were then selected as phenotypic traits associated with PRR resistance. Genetic analysis of these traits was conducted which identified quantitative trait loci (QTL). Additionally, these hydroponic traits and a set of previously published plant survival traits obtained from multiple PRR field experiments were combined in a model-based correlation analysis. The results suggest that the underlying genetic basis for plant survival during PRR infection within hydroponics and field disease environments is linked. The QTL *QRBprrkms03* and *QRBprrck03* on chromosome 4 identified for the traits KME-survival and canker length, respectively, correspond to the same region reported for PRR resistance in a field disease experiment.

**Conclusion:**

A hydroponics based screening system will facilitate reliable and rapid screening in both small- and large-scale experiments to study PRR disease in chickpea. It can be applied in chickpea breeding programs to screen for PRR resistance and classify the virulence of new and existing *P. medicaginis* isolates.

## Background

Chickpea (*Cicer arietinum* L.) is an important legume crop with high nutritional value, mostly cultivated in arid and semi-arid regions of the world. Globally, Australia is the second largest chickpea producing country after India [1]. In Australia, chickpea is mainly grown in northern New South Wales and southern Queensland. Annually, in Australia approximately 90% of the chickpea produced is exported to developing countries like India and the subcontinent where demand exceeds the supply. Chickpea is grown as a rotational crop for its ability to fix atmospheric nitrogen through symbiotic fixation [2]. Susceptibility to soil borne pathogens is a major constraint to the expansion of chickpea production in Australia.

Phytophthora root rot (PRR) caused by an oomycete *Phytophthora medicaginis* E. M. Hansen and D. P. Maxwell is an economically important soil borne disease, causing significant yield loss in the major chickpea growing regions of Australia [3]. The occurrence of the disease is mainly reported in regions of high rainfall, poorly drained soils or following periods of prolonged soil saturation. PRR costs up to $8.2 million per year to chickpea growers in Australia, indicating the need to develop genetic or management solutions [4].

The pathogen *P. medicaginis,* survives as thick-walled oospores in soils of heavy texture, infected plant tissues over a long time period. During favourable conditions such as flooding from either irrigation or following a rainfall event, oospores develop into motile zoospores and are released into the soil. These zoospores swim towards the root of the susceptible host plant and on reaching the root surface, germinate to produce hyphae which invade the roots thus enabling further cycles of infection to occur in the host plant [5]. These zoospores are capable of swimming only a few millimetres and consequently long-distance dispersion of PRR infection is a result of physical movement of soil and water contaminated with oospores during flooding, irrigation or by machinery [6].

*P. medicaginis* has been reported to infect both lucerne and chickpea [7]. The pathogen can infect chickpea at any stage of plant development. The symptoms of PRR in chickpea include seed decay at germination, the decay of lateral and tap roots, defoliation from ground up, chlorosis, and wilting of the entire plant leading to plant death. Dark brown to black lesions (canker) often girdle the taproots of PRR-infected chickpea plants and result in plants being easily dislodged from the soil. In young plants, lesions can extend up the stem above ground level. When there is mild infection, the affected plant recovers by producing new roots from the upper part of the taproot [3].

Once the plant is infected there is nothing that a grower can do to manage the loss from PRR. Metalaxyl-based seed dressings are used before infection, but they are expensive and can only provide protection for 6 to 8 weeks. The only effective way to minimise the incidence of the disease is through pre-sowing decisions and assessment of disease risk for individual paddocks. Breeding for resistance is the desired option to control PRR in chickpea. Moderate field resistance has been identified in a chickpea landrace ICC11870 [3] and the Australian chickpea breeding program has incorporated this resistance into a range of cultivated *C. arietinum* varieties such as Yorker. Furthermore, a high level of resistance has been identified in a wild relative of chickpea (*C. echinospermum*) [3] and has been incorporated into a *C. arietinum* background to generate interspecific hybrids. These chickpea genotypes have been used as resistant parents to develop both intraspecific and interspecific recombinant inbred line (RIL) mapping populations within the Australian chickpea breeding program for the genetic analysis of PRR resistance in chickpea [8]. Genomic regions associated with PRR resistance were identified in a field-based study conducted in three target environments, classified as providing low, moderate and high disease pressure [8]. Additionally, the study reported independent sources of PRR resistance in cultivated and wild *Cicer* species. However, the molecular mechanisms underlying PRR resistance in chickpea are not yet clearly understood.

In order to study the molecular mechanisms underlying the plant response to PRR infection, there is a need to establish controlled environment methods that provide control over the precise timing of infection and consistent inoculum distribution across multiple genotypes. Knights et al. [3] developed a soil-based cup method to screen several wild *Cicer* species for PRR resistance in a greenhouse. Seedlings were grown individually in plastic cups containing 10% (w/w) soil-sand and inoculated with *P. medicaginis* oospores. The seedlings were subjected to repeated cycles of flooding with water (40 h) and draining (56 h), to induce the development of zoospores from the oospore inoculum and initiate PRR infection. Plants were scored for survival time after inoculation. The study compared findings from the cup method with field experiments and reported a significant discrepancy in PRR resistance rankings of genotypes [3]. These greenhouse experiments failed to reveal differences in PRR resistance in the chickpea genotypes used as check genotypes. The reasons for the variable results in the greenhouse experiment were explained in the context of the findings of Dale and Irwin [9]. They suggest resistance to *P. medicaginis* that is effective in chickpea roots may not be expressed if the infection occurred through stomata near the soil surface. Fundamentally, greenhouse seedling tests differ in many ways from the field disease experiments that typically cover more growth stages of chickpea and therefore may be less sensitive in resolving differences in resistance between genotypes [3]. This suggests that the choice of inoculum and the methodology used in these seedling tests has a major influence in disease distribution and in the expression of resistance in a given host plant. In other phytophthora pathosystems, such as for soybean, PRR resistance is studied using tests that can cause localized infection in wounded and non-wounded cotyledons or roots [10–12], layer tests [13–15] and rice screen tests [16]. The application of these methods is limited to a smaller number of genotypes and therefore not amenable to screening of breeding populations. Thus, there is a need for the development of an efficient, reproducible, higher throughput phenotyping approach to deriving a quantitative trait that is closely associated with the resistance phenotype expressing the same resistance observed in natural disease environment.

Given the epidemiology of the pathogen and specificity of the pathosystem, the main objective of this research was to develop a hydroponics based screening system using zoospore inoculation to control the timing and rate of infection that could be applied in breeding. An important specific objective was the derivation, quantification and analysis of PRR resistance related traits from the developed screening system. This included the individual and co-analysis of PRR traits using modern linear mixed modelling approaches that appropriately quantified environmental variation and accurately estimated the underlying genetic potential of PRR resistance across each genotype. Extended formulations of these models were then used to conduct QTL analysis and identify genomic regions associated with PRR resistance that were then compared to published results from recent PRR field trial research. A final objective was to conduct a multi-trait multi-environment analysis and directly assess the genetic connection between PRR traits derived from hydroponic screening and previous published plant survival traits obtained from field experiments.

## Results

### PRR symptom development in hydroponics based *in planta* infection system

**Fig. 1:**
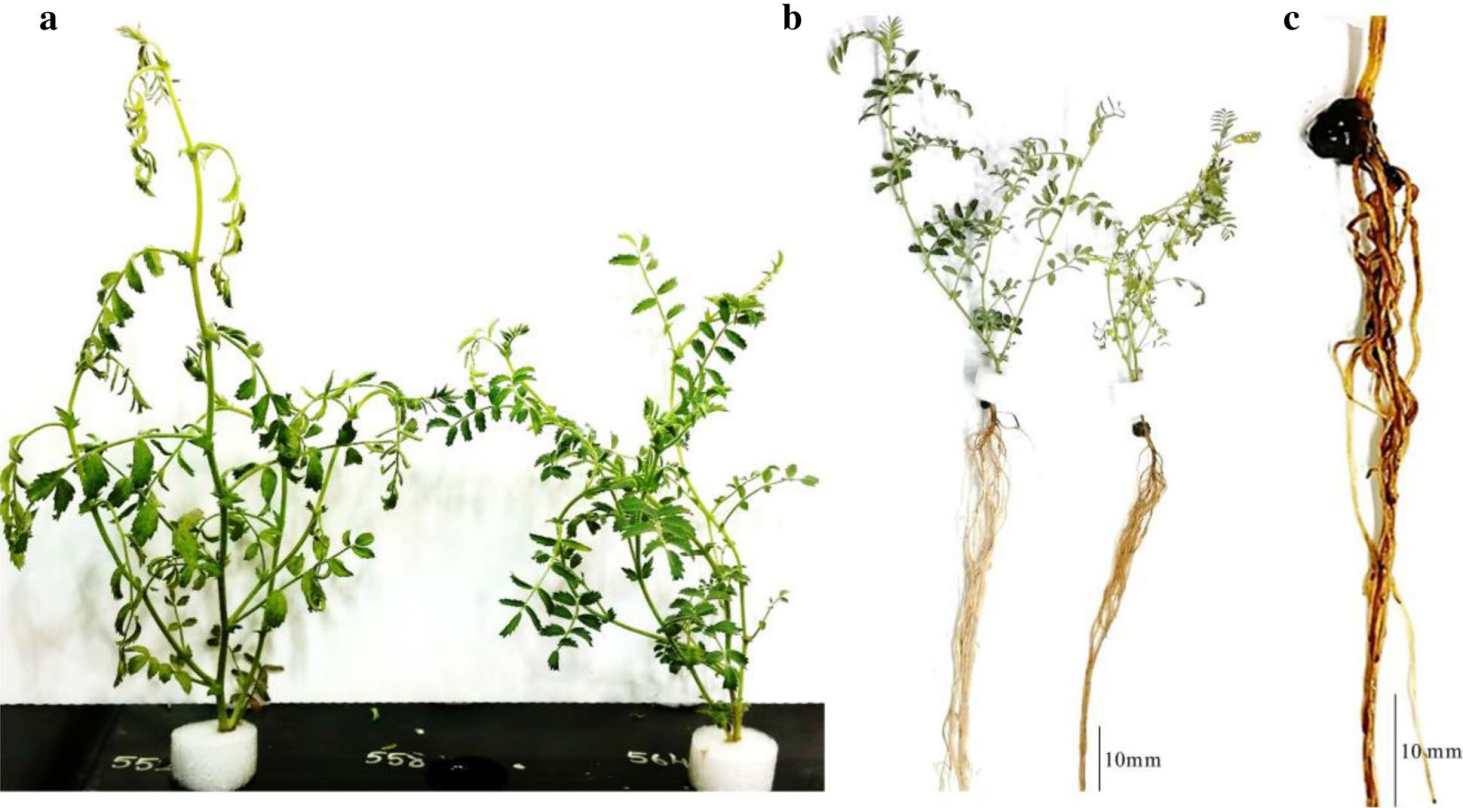
Phenotypic variation for PRR resistance in chickpea grown in hydroponics at 9 days after inoculation with *P. medicaginis* zoospores. **a** Wilting symptoms (04067-81-2-1-1 on left, Rupali on right) chickpea genotypes grown in hydroponics at 9 days after inoculation with *P. medicaginis* zoospores. **b** Root symptoms (04067-81-2-1-1 on left, Rupali on right). **c** Lateral and tap root death in Rupali

**Fig. 2:**
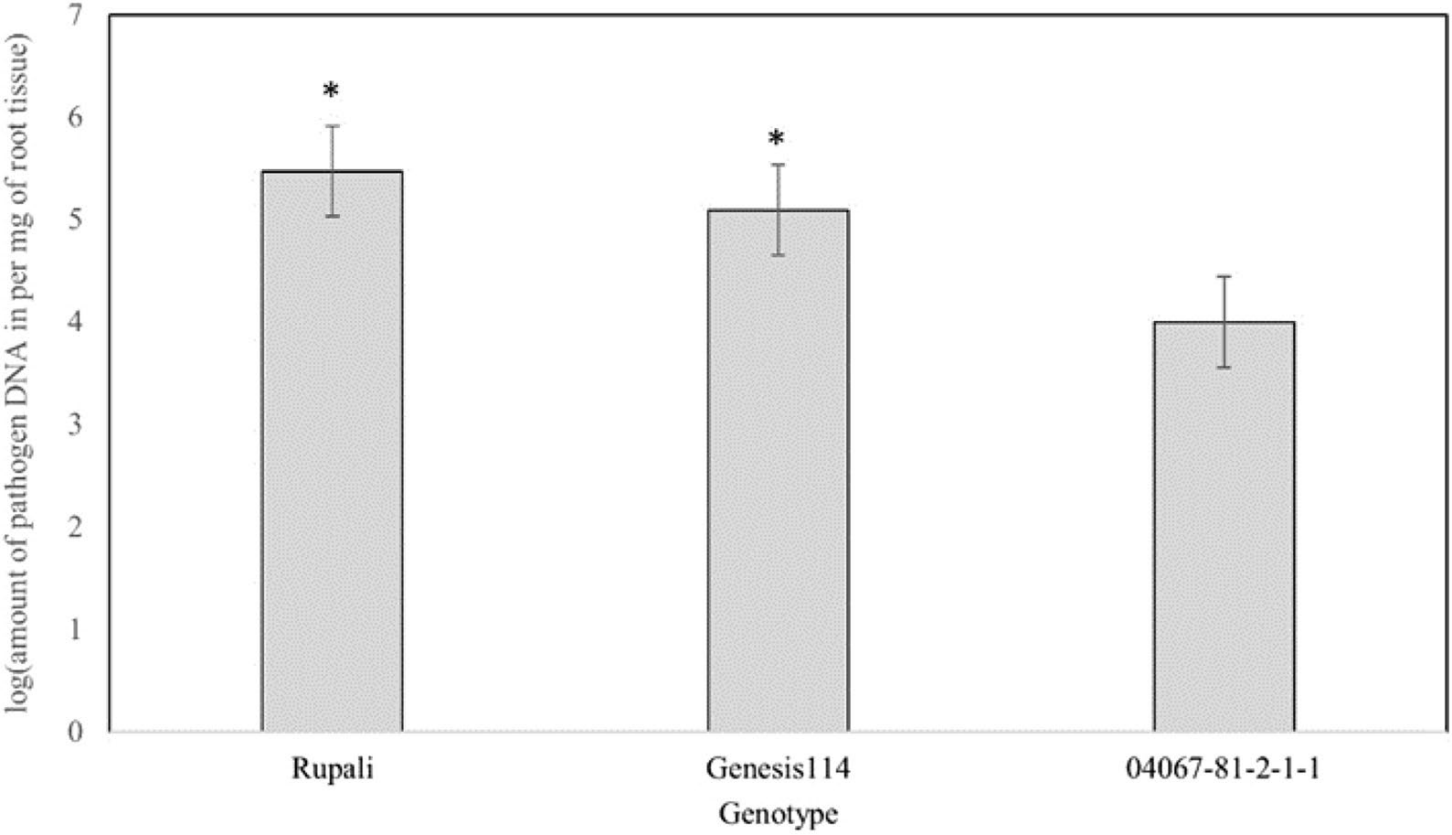
Molecular quantification of PRR DNA in roots of chickpea genotypes with known levels of PRR resistance. The log-transformed values of the amounts of *P. medicaginis* DNA determined by a TaqMan MGB assay is presented. Error bars represent the standard error of the mean of six biological replicates. Asterisks indicate significant differences compared to PRR-resistant 04067-81-2-1-1 at the 5% (*P* < 0.05) significance level

Symptoms characteristic of PRR such as brown to black lesions and wilting were observed in the PRR susceptible chickpea variety Rupali at 9 days after inoculation (Fig. 1). The PRR resistant breeding line 04067-81-2-1-1 showed no symptoms and remained healthy following inoculation until the termination of the experiment. The presence of *P. medicaginis* in chickpea roots harvested from the varieties Rupali, Genesis 114 and breeding line 04067-81-2-1-1 was confirmed and quantified using a TaqMan MGB assay specific for *P. medicaginis* DNA. The amount of *P. medicaginis* DNA was found to be relatively high in the PRR-susceptible variety Rupali and in the moderately susceptible variety Genesis 114 compared to the PRR-resistant breeding line 04067-81-2-1-1 (Fig. 2; *P *< 0.05). *P. medicaginis* DNA quantification in each chickpea genotype was in accordance with observations of PRR symptom development and with known levels of PRR resistance of these genotypes from field experiments and trials.

### Response of RIL mapping populations to *P. medicaginis* and disease progression in hydroponics PRR screening system

To understand disease progression in the two RIL populations, the interval time of death and the canker length at the close of the experiment were recorded for each plant. From the known time of death, KME-survival probability was then derived for each plant to use for exploratory and quantitative analyses. The PRR moderately resistant parent of the YG population, Yorker, showed lower KME-survival values and short canker length while the PRR susceptible parent, Genesis 114, showed a slightly higher KME-survival value and a longer canker length (Table 1). Similarly, in the RB population, the breeding line 04067-81-2-1-1 which is known to exhibit higher PRR resistance showed a lower KME-survival value and a short canker length compared to the PRR susceptible parent Rupali. The population means for YG and RB for the KME-survival were 0.475 and 0.484, respectively, while the population mean and values for the canker length trait (Table 1) were found to be in general high for the YG population compared to that of the RB population.

**Table 1:** Parental and population means for the KME-survival and canker length in each RIL population screened for PRR resistance in hydroponics

Trait	Parental mean		RIL population		
	Yorker	Genesis 114	Mean	Range	Heritability H^2^
KME-survival	0.334	0.457	0.475	0.058–0.995	0.427
Canker length (mm)	81.42	101.63	89.38	0–158.0	0.159

Trait	Parental mean		RIL population		
	Rupali	04067-81-2-1-1	Mean	Range	Heritability H^2^
KME-survival	0.676	0.243	0.484	0.243–0.943	0.448
Canker length (mm)	23.437	4.559	8.300	0–50.0	0.421

**Fig. 3:**
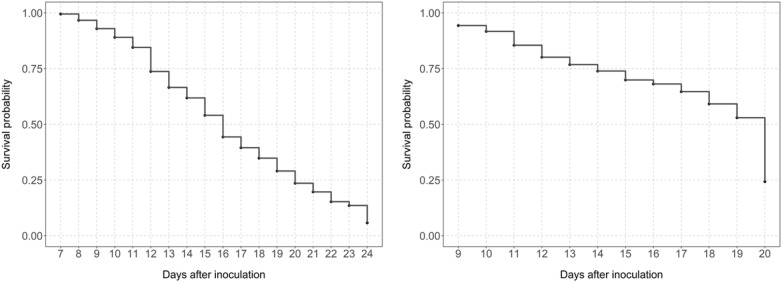
KME-survival showing progress of PRR disease caused by *P. medicaginis* in YG (left) and RB (right) chickpea RIL mapping populations grown in hydroponics system

To visualise the progression of PRR disease over the duration of the experiments the KME-survival were plotted, based on the initial observation of PRR symptoms after inoculation until the termination of the experiment (Fig. 3). The termination of the experiment was based on the death of the PRR susceptible parental genotype included in each of the RIL mapping population. In the YG population, it was marked by the death of the PRR susceptible variety, Genesis 114 and in the RB population it was marked by the death of PRR susceptible variety Rupali. For the YG population, there was a linear reduction in survival probability over time and survival approached zero at the termination of the experiment. In the RB population, this linear reduction in survival probability was less pronounced for most of the experiment i.e. 18 days post inoculation but then the survival probability sharply reduced from 0.5 to 0.25 in the final interval before the experiment terminated.

**Fig. 4:**
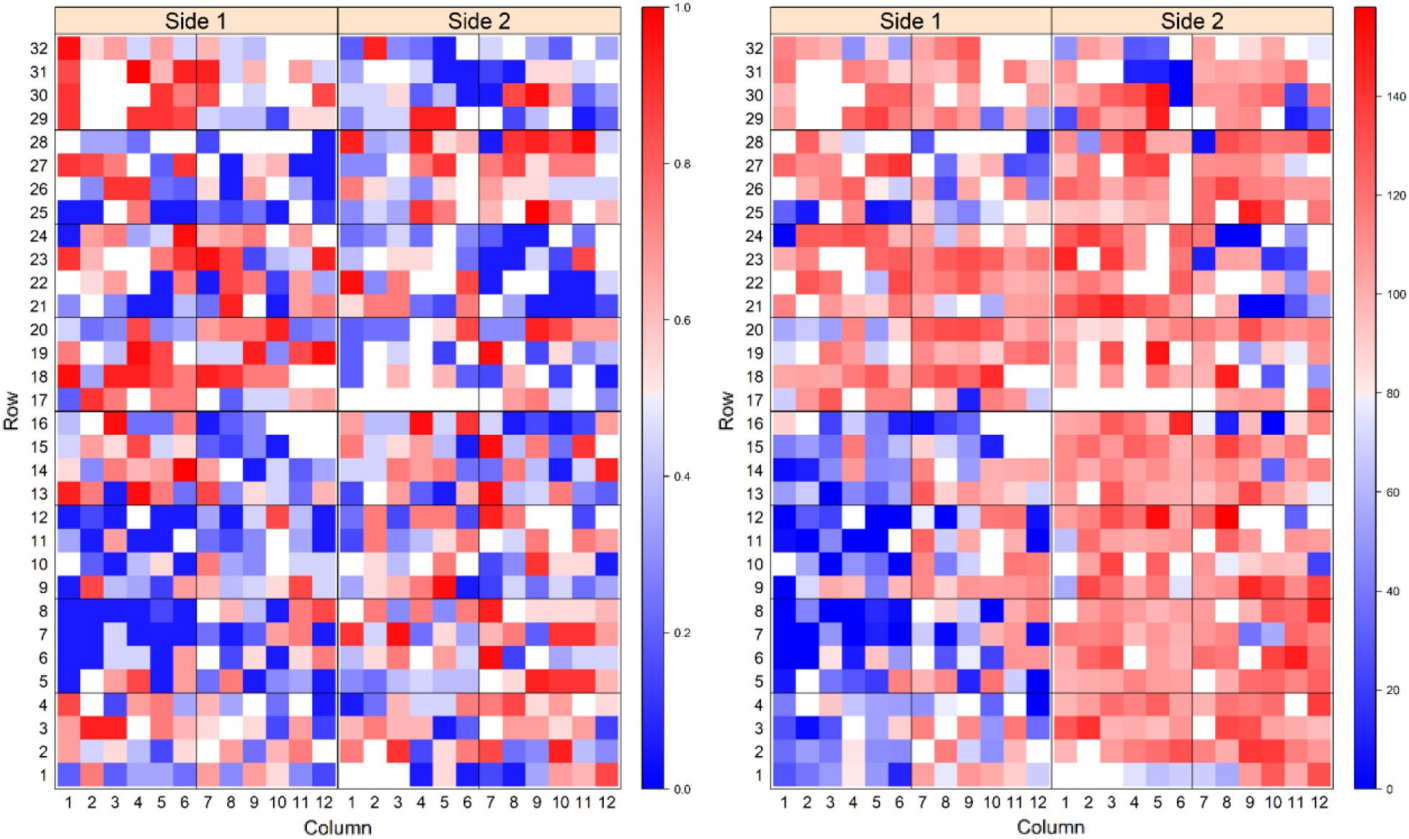
Heat map plots showing prevalence of PRR disease at the close of the experiment in the YG population based on the traits KME-survival (left) and canker length (right) data. For the KME-survival heat map blue areas indicate longer time of survival and red areas indicate short survival time. Blue areas on the heat map plot for canker length indicate no or negligible canker and red areas indicate longer length of canker

**Fig. 5:**
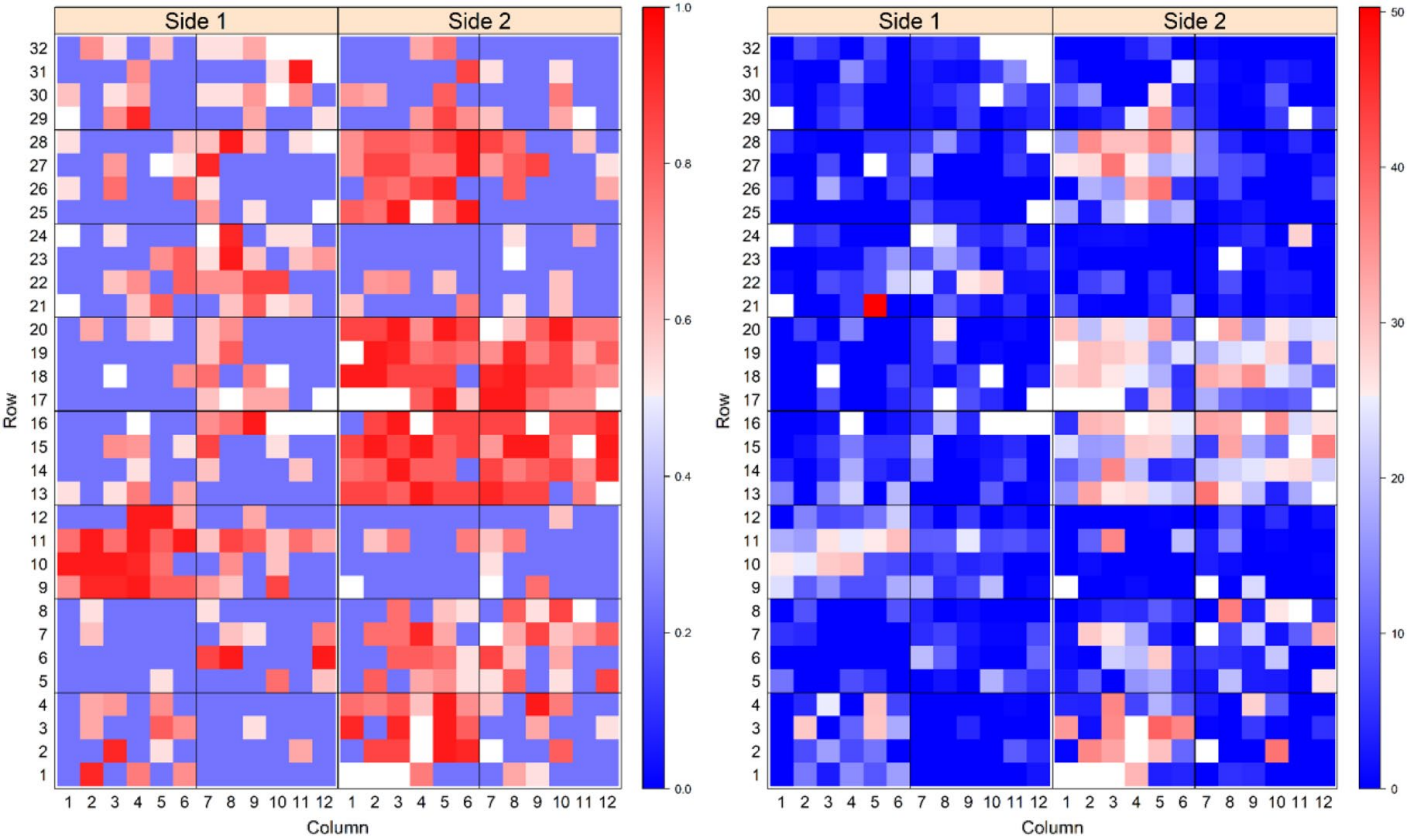
Heatmap plots showing prevalence of PRR disease at the close of experiment in RB population for traits KME-survival (left) and canker length (right) data. For the KME-survival heat map blue areas indicate longer survival time and red areas indicate shorter survival time. Blue areas on the heat map plot for canker length indicate no or negligible canker and red areas indicate longer length of canker

To visualise the spatial progression of PRR disease across the experiment, heat maps were plotted for each RIL population to graphically display the KME-survival and canker length at the termination of the experiment (Fig. 4 and Fig. 5). In the heat maps displaying KME-survival, blue areas indicate low KM estimate values and consequently higher resistance to disease while redder areas indicate high KME-survival and plants with increased susceptibility to the disease (Figs. 4 and 5). Similarly, in the heat map displaying the canker length of plants, blue areas indicate negligible lesion length suggesting high resistance to PRR and redder areas indicate longer lesion length and increased susceptibility to PRR (Figs. 4 and 5). In the RB population, lower KME-survival and shorter relative canker lengths (bluer areas in Fig. 5) were more pronounced compared to the YG population, using the same inoculum concentration.

### Relationship between phenotypic traits measured in hydroponics

**Fig. 6:**
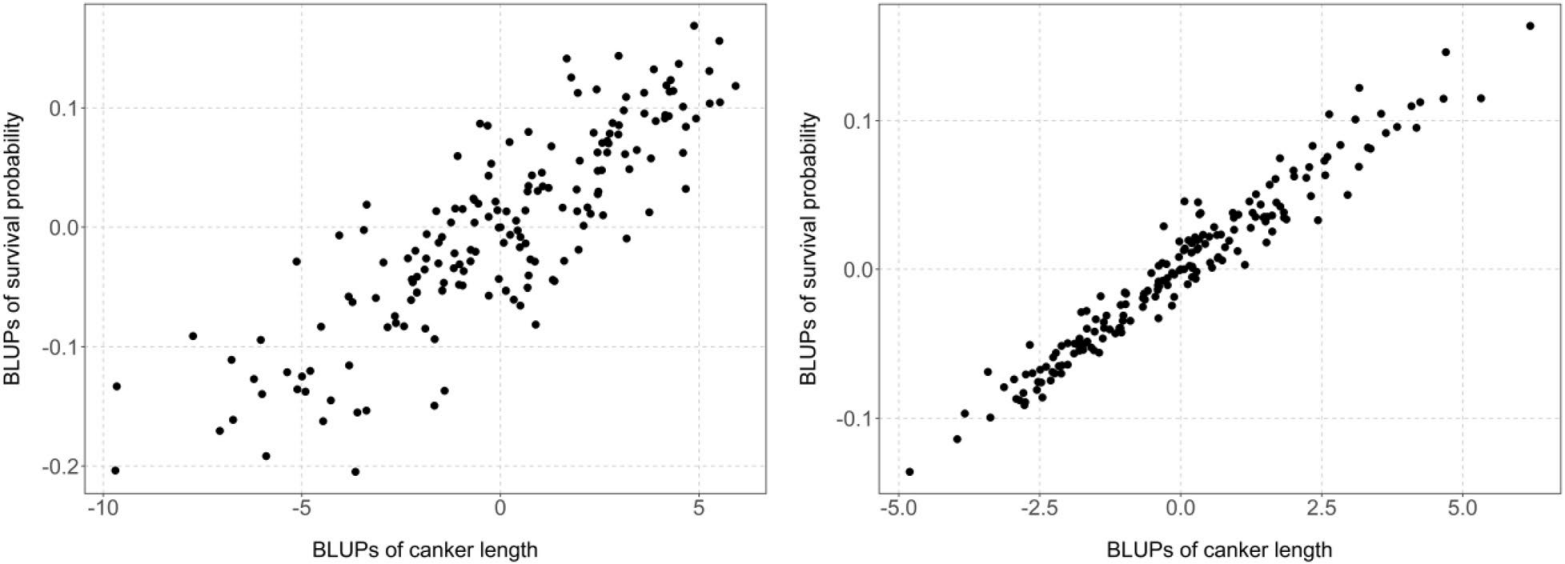
Correlation plots of the BLUPs for KME-survival and canker length extracted from the bivariate linear mixed model. YG (left) and RB (right) RIL mapping populations

For each mapping population the BLUPs of the KME-survival and canker length were extracted from the BLMM. A strong linear genetic relationship exists between the traits (Fig. 6) in each population with model based genetic correlation estimates of 0.642 and 0.917 in YG and RB mapping populations (Table 3), respectively. The estimated broad sense heritability values (H^2^) values for each trait (Table 1) were found to be adequate in the interspecific mapping population RB. Heritability for KME-survival was also found to be adequate in the YG population but reduced for canker length. Despite the varied heritability values, the high estimated genetic correlation between the two traits observed in both the intra and interspecific mapping populations indicates a similar genetic expression of the traits in the presence of PRR disease.

### QTL analyses for YG population

QTL analysis conducted using whole genome average interval mapping (WGAIM) identified genomic regions associated with PRR resistance for KME-survival obtained from hydroponics phenotyping of the YG RIL population (Table 2). For the KME-survival, one major QTL *QYGprrkms01* on chromosome 7 explaining 16.3% of genetic variation was identified to be associated with PRR resistance. No QTL were identified for canker length in the YG population, suggesting inadequate genetic variation. This lack of genetic variation is also indicated by a reduced heritability value (H^2^) of 0.159.

**Table 2:** QTL associated with the traits KME-survival and canker length for PRR resistance in YG and RB RIL mapping populations screened in hydroponics experiment

Population	Trait	Chr.	QTL name	Interval	Distance (cM)	Size^a^	p.value	%Var	LOD
YG	KME-survival	C7	*QYGprrkms01*	*9001SNP7*-*31: 9001SNP7*-*32*	0.0–1.22	− 0.271	0.000	16.3	2.668
RB	KME-survival	C2	*QRBprrkms01*	*RB*-*SNP2*-*32: RB*-*SNP2*-*31*	1.5–16.49	0.141	0.001	8.1	2.199
		C3	*QRBprrkms02*	*RB*-*SNP3*-*9: RB*-*SNP3*-*97*	54.66–58.84	0.153	0.001	9.6	2.627
		C4	*QRBprrkms03*	*RB*-*SNP*-*79(C): RB*-*SNP*-*72(C)*	80.53–81.7	0.176	0.000	12.6	2.881
		C6	*QRBprrkms04*	*RB*-*SNP6*-*183(C): RB*-*SNP6*-*417(C)*	74.66–75.82	0.259	0.000	27.5	7.366
		C7	*QRBprrkms05*	*RB*-*SNP7*-*30: RB*-*SNP*-*scf136(C)*	95.25–112.92	− 0.188	0.000	14.5	3.634
	Canker length	C2	*QRBprrck01*	*RB*-*SNP2*-*6: RB*-*SNP2*-*7*	77.49–79.04	1.031	0.000	8.5	2.403
		C3	*QRBprrck02*	*RB*-*SNP3*-*100(C): RB*-*SNP3*-*105*	62.34–63.71	1.629	0.000	21.2	5.902
		C4	*QRBprrck03*	*RB*-*SNP*-*79(C): RB*-*SNP*-*72(C)*	80.53–81.70	1.366	0.000	14.9	4.378
		C6	*QRBprrck04*	*RB*-*SNP*-*scf200(C): RB*-*SNP6*-*322(C)*	83.31–84.35	1.274	0.000	12.9	3.505
		C7	*QRBprrck05*	*RB*-*SNP7*-*30: RB*-*SNP*-*scf136(C)*	95.25–112.92	− 1.305	0.000	13.6	2.859

QTL interval names with (C) indicate several co-locating markers at the loci

^a^Positive and negative values indicate that Yorker and Genesis 114 alleles increased the phenotypic values in the YG population, and 04067-81-2-1-1 and Rupali alleles in the RB population, respectively

### QTL analyses for RB population

In the interspecific mapping population RB, QTL analyses identified genomic regions associated with PRR resistance for both hydroponic traits (Table 2). For KME-survival, three major QTL on chromosomes 4, 6 and 7 associated with PRR resistance were found to be significant accounting for 12.6%, 27.5% and 14.6% of genetic variation, respectively. Two minor QTL on chromosomes 2 and 3 were also identified. Similarly, for canker length, four QTL on chromosomes 3, 4, 6 and 7 associated with PRR resistance were identified accounting for 21.2%, 14.9%, 12.9% and 13.6% of genetic variation, respectively. For the QTL identified on chromosomes 3, 4 and 6, the resistance source is contributed by the high PRR resistant parental genotype 04067-81-2-1-1 derived from *C. echinospermum*. The genomic regions *QRBprrkms03* and *QRBprrck03* on chromosome 4 and *QRBprrkms05* and *QRBprrck05* on chromosome 7 co-locate, confirming a genetic inter-relatedness between the hydroponic traits in association to PRR resistance. *QRBprrkms02* and *QRBprrck02* on chromosome 3 and *QRBprrkms04* and *QRBprrck04* on chromosome 6 identified for the traits KME-survival and canker length were found to be in similar genetic intervals.

### Relationship between phenotypes in hydroponics experiment and field experiments

The estimated genetic correlation matrix was extracted from the fitted MVE-LMM and broadly shows a negative relationship between the hydroponics phenotypic traits and the plant survival traits collected from the multiple field environments (Table 3) of Amalraj et al. [8]. For the RB population, there were strong negative estimated genetic correlations between the plant survival trait from the 2014 rainfed field experiment and the two traits measured in hydroponics (− 0.781 for the KME-survival trait and − 0.853 for canker length trait. This indicates, that in the presence of PRR disease, the underlying genetic basis for plant survival in the controlled environment is linked to plant survival in the field. The strength of these negative correlations was reduced between the hydroponics traits and the plant survival trait measured in 2015 rainfed and 2015 irrigated field experiments (Table 3). In the intraspecific YG population, there were consistently lower estimated genetic correlations between hydroponics traits and plant survival traits measured in all field experiments. This may be due to reduced range in the levels of PRR resistance between the parental genotypes Yorker and Genesis 114 used to develop the YG mapping population [8].

**Table 3:** Estimated genetic correlation from the fitted multi-environment model for KME-survival and canker length in each of the two RIL mapping population

	Experiment	2014 rainfed	2015 rainfed	2015 irrigated	KME-survival
YG					
Survival index	2014 rainfed	0.282			
	2015 rainfed	0.442	0.442		
	2015 irrigated	0.388	0.819	0.454	
KME-survival	Hydroponics	0.009	0.079	0.135	
Canker length	Hydroponics	− 0.165	− 0.221	− 0.101	0.642
RB					
Survival index	2014 rainfed	0.883			
	2015 rainfed	0.890			
	2015 irrigated	0.833	0.921		
KME-survival	Hydroponics	− 0.781	− 0.557	− 0.662	
Canker length	Hydroponics	− 0.853	− 0.597	− 0.705	0.917

## Discussion

In this study, we have developed a scalable, hydroponics based phenotyping method for PRR disease screening, using zoospore inoculation. Results from the genetic analyses of the hydroponic phenotypic traits as well as combined hydroponic and field model-based correlation analysis, show there is an underlying genetic connection between hydroponic PRR survival traits derived using this newly developed screening method and field PRR survival traits from previously published field-based experiments. This method can overcome the limitations imposed by current PRR screening systems by reproducing the natural course of PRR infection, wherein zoospores can freely access the roots of the host chickpea plants. The use of zoospore as the inoculum is considered to be beneficial as it can immediately infect host plants in the system thereby eliminating the conditions such as flooding and draining needed for the development of phytophthora pathogen to cause PRR infection. The flooding and draining methodology can potentially introduce irregularity in the PRR disease development across a large number of genotypes that could ultimately lead to inappropriate expression of resistance in host chickpea plants. Thus, the zoospore inoculum is considered to be advantageous in maintaining the virulence pattern of the pathogen (as reported for, *P. sojae* isolates [17]), which is essential for the appropriate expression of host resistance.

An important factor to be considered when assessing PRR resistance in controlled systems is the choice of isolate used [18]. The chosen isolate should have compatible interaction with all chickpea genotypes included in the study, to generate an appropriate disease pressure. In an unpublished (Sean Bithell, personal communication) inoculated glasshouse experiment for the testing of phytophthora isolates on the parental chickpea genotypes of the two RIL mapping populations, the isolate 1129-1 showed a pathogen–host interaction profile (in terms of plant mortality) similar to that of a mix of 10 *P. medicaginis* isolates used in field disease experiments and trials within the breeding program. For this reason, the isolate 1129-1 (DAR 66075) collected from Yetman, New South Wales, Australia [19], which has been reported to infect chickpea and *Hedysarum* spp., was used in this study. Initial pilot-scale testing confirmed the compatible interactions of the chickpea genotypes Rupali, Genesis 114 and breeding line 04067-81-2-1-1 with the *P. medicaginis* isolate 1129-1. These genotypes are the parents of two RIL mapping populations, RB and YG, developed for genetic studies of chickpea PRR responses as described in Amalraj et al. [8].

To test the efficiency of the hydroponics screening system to cause PRR infection in chickpea, qualitative and quantitative assessments were made of the responses of three genotypes with contrasting PRR disease resistance levels in the field. The hydroponics assay showed good discrimination between these genotypes (Table 1 and Fig. 2). Molecular quantification of the amounts of *P. medicaginis* DNA in chickpea roots using a previously developed TaqMan MGB assay [20] for *P. medicaginis* provided quantitative evidence of the phenotypic responses of the host plant genotypes to PRR infection (Fig. 2). The TaqMan MGB assay is a real-time fluorescent PCR assay using a set of specific primers and a fluorochrome-labelled probe. Primers used were developed from a sequence-characterized DNA marker (SCAR) that is specific only for *P. medicaginis* [21]. The assay demonstrated *P. medicaginis* infection in the zoospore-inoculated plants, and ranked disease severity in the chickpea genotypes similarly to visual assessments of plant survival and stem canker length. However, for practical reasons such as time involved and assay cost, it was determined that TaqMan DNA assay would not be the preferred choice to phenotype large number of breeding populations.

Scaling up of the hydroponics system allowed for high-throughput screening of a large number of genotypes to evaluate PRR resistance with an aim to identify linked quantitative traits that could potentially be used to assist selection in chickpea breeding. We screened the two RIL mapping populations, RB and YG, in consecutive experiments. Disease assessments were made during early stages of disease progression because older chickpea plants are able to survive by generating new roots from the upper main tap root [6].

Quantitative disease resistance (QDR) conditioned by a few to many genes is the preferred type of resistance in most crop breeding programs because of its increased durability [22]. However, phenotyping for quantitative resistance is challenging due to the polygenic nature of the phenotype and requires accurate methods for disease assessment with adequate replication. For some pathosystems, such as *P. medicaginis*-chickpea, where there is no conclusive evidence for race-based resistance, breeding for QDR is the only option to limit the impact of the disease and prevent yield loss. This involves identification of QTL associated with disease resistance, using accurate measurements of the disease to facilitate marker-assisted selection. The hydroponics phenotyping experiment in this study provided a basis for objectively measuring and deriving two quantitative traits linked to PRR resistance in chickpea: KME-survival and canker length. KME-survival and canker length data were then used in subsequent phenotypic and genetic analysis.

KME-survival and canker length were highly correlated in both the intraspecific (YG) and interspecific (RB) RIL mapping populations (Fig. 6). This is also evident from the co-location of QTL *QRBprrkms03*, *QRBprrck03* on chromosome 4 and *QRBprrkms05*, *QRBprrck05* on chromosome 7 in the RB population. The co-location of these QTL associated with PRR resistance suggests that the same genomic region(s) control the expression of two traits. In the RB population, the major QTL *QRBprrkms03 and QRBprrck03* on chromosome 4 (*RB*-*SNP*-*79(C): RB*-*SNP*-*72(C))* was also reported to be associated with PRR resistance in chickpea grown under rainfed field conditions [8]. Similarly, the major QTL *QRBprrck04* on chromosome 6 identified in this study is similar to a genetic interval *QRBprrsi04* reported across multiple field experiments from the field-based genetic study [8]. In both cases the resistance source is from the parental genotype 04067-81-2-1-1, a backcross derivative line from *C. echinospermum*.

The similarity of PRR QTL for the traits phenotyped in hydroponics and the field provides evidence of genetic similarity in the plant response to this disease across different environments. This was further supported by model-based correlation analyses, which for the RB RIL population showed a strong negative correlation between KME-survival and canker length from hydroponics and the plant survival trait (survival index) from the field-based genetic study described in Amalraj et al. [8] (Table 3). The genetic correlation between these traits indicates that genetic factors underlying the loci associated with PRR resistance in chickpea may be present in a similar location of the same chromosome. It also suggests that the host–pathogen interaction pattern of the *P. medicaginis* isolate 1129-1 used in this experiment is similar to that of the mix of *P. medicaginis* isolates used in the field based genetic study [8]. In YG population, the correlation was weak, and this could be because the PRR disease pressure prevailing in the hydroponics experiment seemed to be high for this intraspecific population. Moreover, from the field-based experiment it was shown that Yorker, the resistant parent in the YG population was classified to be moderately resistant to PRR. These moderately resistant chickpea plants become highly susceptible under high inoculum loads and when conditions are favourable for the development of the disease. This is in agreement with YG field-based disease experiments described in Amalraj et al. [8], which showed consistently low to moderate broad sense heritability values across the conditions of low, moderate and high disease pressure. The similar performance of YG in the field and controlled environment experiments exhibiting moderate PRR resistance indicate that a stronger source of PRR resistance is present in RB, where the resistance source is derived from *C. echinospermum*. For PRR, the strategy of using resistance genes/alleles from wild chickpea relatives in breeding has shown it can provide better adaptation under high disease pressure than the currently available variants in the cultivated gene pool. This strategy of crop improvement has been broadly supported recently with a substantial increase in the development of genomic resources and the development of pre-breeding populations in wild relatives of *Cicer* for use in chickpea breeding [23]. In this study, the differences in heritability of the PRR related hydroponic traits obtained from screening RB and YG RIL populations further highlights the differences in genetic complexity of traits identified between wild and cultivated *Cicer* species.

While the hydroponics system described in this study has proven to be reliable for the purpose of studying *P. medicaginis*-chickpea interactions, specific requirements in terms of greenhouse facilities, materials for hydroponics aeration systems for proper oxygenation and nutrient circulation can be modified while maintaining the basic principles conferring its efficiency. The disease pressure to induce the required level of PRR infection can be varied by altering the concentration of compatible *P. medicaginis* zoospores applied as the inoculum. Furthermore, consideration can be given to the use of zoospores of more than one *P. medicaginis* isolate, which could prove to be beneficial in a breeding program when screening large number of diverse genotypes for partial resistance to PRR. The use of zoospore inoculum to cause PRR disease enabled the generation of a uniform disease across the large number of plants grown in hydroponics system. In field disease conditions of PRR, the lesions formed at the base of the chickpea stem may themselves liberate zoospores, forming a secondary source of zoospore inoculum able to reinfect neighbouring chickpeas [24]. Additionally, the use of hydroponics as a phenotyping system enabled the phytophthora zoospores to access both the roots and hypocotyl region of the cotyledon for pathogen invasion. Thus, we report that adopting the right choice of inoculum in suitable plant growth system will facilitate the appropriate expression of virulence and avirulence factors in the pathogen and resistance genes in the host plant under controlled environments. Given that the zoospore is the infecting stage of the phytophthora life cycle, using it as the inoculum of choice can promptly and evenly cause PRR infection in the host plant. In contrast to applying oospores or mycelium slurry, the use of zoospores allows specific control of infection timing which will be beneficial for research aimed at the identification of specific genes or biological processes involved in the temporal plant response to PRR infection. Furthermore, this system proves advantageous for easy sampling of plant tissues like roots free from soil for molecular studies. The similarity in the underlying genetic components of the phenotype traits KME-survival, canker length and survival index using a model-based correlation analysis, support the application of this hydroponic phenotyping method as a viable alternative in breeding to laborious and relatively expensive field-based protocols that are impacted by environmental variation.

## Conclusion

The present study describes a hydroponics screening system to study *P. medicaginis*–chickpea interactions using a zoospore inoculation technique. This system has been validated both qualitatively, based on observation of characteristic PRR symptom development, and quantitatively based on the pathogen DNA quantification in roots, thereby making it suitable to conduct both small- and large-scale experiments for PRR resistance. Two phenotyping traits, plant KME-survival and canker length, used to select for PRR resistance showed a high genetic correlation. QTL analysis and model-based correlation analysis has shown this phenotyping method enables the expression of the same PRR resistance in both the field and under controlled environment. Thus, the application of this method will facilitate current and future efforts in breeding for PRR resistance in chickpea, as well as genetics studies aimed at identifying both PRR resistance genes in chickpea and virulence factors in *P. medicaginis*.

## Methods

### Aim and design of the study

The main objective of this study was to develop a screening system as an efficient phenotyping method under controlled environment to study PRR resistance in chickpea and potentially be applied in breeding. The specific objective included the analysis of PRR resistance related traits using linear mixed modelling approach to quantify the genetic potential underlying each chickpea genotype and use it to identify QTL associated with PRR resistance in chickpea. Further, a multi-trait multi-environment analysis was used to assess the genetic connection between PRR traits derived from the hydroponics system and previously published plant survival traits obtained from field experiments.

### Plant and fungal material

One each of an intraspecific and interspecific F_6_ derived RIL mapping population was used in this study. The intraspecific F_6_ derived RIL population, herein referred to as YG, consisted of 192 RIL individuals derived from a cross between the moderately PRR resistant chickpea variety Yorker (pedigree: 8507-28H/946-31) and the moderately PRR susceptible variety Genesis 114 (pedigree: FLIP91-150C/FLIP90-124C//S9231). The interspecific F_6_ derived population, herein referred to as RB, consisted of 212 RIL individuals derived from a cross between a highly PRR resistant breeding line 04067-81-2-1-1 (a backcross derivative from *C. echinospermum* pedigree: Howzat/ILWC 245//99039-1013) and a PRR susceptible chickpea variety Rupali (pedigree: FLIP84-15C/ICCV88516//Amethyst). The *P. medicaginis* isolate 1129-1, which was recovered from the field in Yetman, New South Wales was used to infect chickpea.

### Hydroponics based *in planta* infection

An *in*-*planta* infection method to screen chickpea for PRR resistance was developed using hydroponically-grown seedlings inoculated with *P. medicaginis* zoospore suspension culture. Three chickpea genotypes were utilised in the initial pilot scale study: PRR susceptible Rupali, moderately susceptible Genesis 114 and the highly PRR resistant breeding line 04067-81-2-1-1 (a backcross derivative from *C. echinospermum*). The experiment was conducted in a temperature-controlled growth room at the University of Adelaide, Waite campus, South Australia, Australia at 20/14 ± 2 °C day/night temperatures with a 16 h photoperiod. Covered plastic pots (4.5 L) were used to grow plants in continuously aerated nutrient solution. The composition of the full-strength nutrient solution, in deionized water, was (mM): 5.0 Ca^2+^, 5.0 K^+^, 0.625 NH^4+^, 0.4 Mg^2+^, 0.2 Na^+^, 5.4 SO_4_^2−^, 4.4 NO_3_^−^, 0.2 H_2_PO_4_^−^, 0.1 SiO_3_^2−^, 0.1 Fe-sequestrene, 0.05 Cl^−^, 0.025 BO_3_^3−^, 0.002 Mn^2+^, 0.002 Zn^2+^, 0.0005 Cu^2+^, 0.0005 MoO_4_^2−^ and 0.001 Ni^2+^ [25]. The solution was buffered with 1.0 mM MES (2-[N-morpholino] ethane sulfonic acid) and adjusted to pH 6.5 using KOH. Seeds were washed with commercial bleach (0.042% (w/v) sodium hypochlorite) added to deionized water for 5 min, rinsed twice in tap water and imbibed at 4 °C for 48 h. Imbibed seeds were then germinated on mesh in 10% aerated nutrient solution in the dark for 3 d and seedlings were then transferred to continuously aerated 25% nutrient solution and exposed to light. Each pot had one healthy individual from each genotype and the pots and position of each genotype was set up in a completely randomised block design with six replicates in control (no inoculation) and in treatment (with inoculation). *P. medicaginis* zoospore suspension culture was added to the treatment pots at a concentration of 1.5 × 10^5^ spores mL^−1^. Plants were examined daily after inoculation for PRR symptoms including canker development, chlorosis and wilting/death. The experiment was terminated at 16 days after inoculation and repeated three times.

### Zoospore production

*P. medicaginis* zoospore production was based on a protocol developed for zoospores of *P. sojae* and *P. cajani* [26, 27]. One piece of mycelial mat (5 mm) from a pure culture of *P. medicaginis* isolate 1129-1 was used to inoculate 100 mL of sterile 20% (v/v) V8 broth containing 0.2% (w/v) calcium carbonate in a 250 mL conical flask and incubated in the dark at 25 °C for 72 h. The V8 broth was then decanted and replaced with sterile deionized water, which was immediately decanted and replaced with salt solution (per litre: 0.294 g CaC1_2_.2H_2_0, 0.247 g MgSO_4_.7H_2_0, and 0.075 g KC1). The mycelial culture was washed five times with the salt solution at 30-minute intervals with incubation at 20 °C, and finally incubated in 25% nutrient solution for about 20 h in the dark. The development of zoospores was confirmed by visualisation using an optical microscope. Mycelial growth was removed by centrifugation at 100 x g for 5 minutes and the zoospores were harvested in the nutrient solution. The concentration of the zoospores was determined using a haemocytometer. An average concentration of 8 × 10^5^ zoospores per mL was obtained for the *P. medicaginis* isolate.

### Molecular quantification of *P. medicaginis* in chickpea roots grown in in planta infection system

The roots of control and treated chickpea varieties Rupali, Genesis114 and breeding line 04067-81-2-1-1 were harvested 16 h after inoculation, rinsed thoroughly with tap water, patted dry on clean paper towel, weighed and snap-frozen in liquid nitrogen. Frozen roots were freeze-dried and the amount of *P. medicaginis* DNA in chickpea roots quantified using a TaqMan MGB assay developed for *P. medicaginis* at the South Australian Research and Development Institute (SARDI) [20, 28, 29].

### Phenotyping two RIL mapping populations (YG and RB) for PRR resistance

Phenotyping experiments to screen two RIL mapping populations were conducted consecutively in the same controlled-environment growth room that was used for the pilot experiment. Seeds from each RIL population, parental genotypes and three check varieties (PBA Slasher, PBA HatTrick and PBA Boundary) were used. Surface-sterilised and imbibed seeds were germinated in plastic pots containing equal volumes of perlite: vermiculite mix covered with aluminium foil. After 7 days the germinated seedlings were transplanted into a scaled-up hydroponics system. Plastic tanks (12.5 L) were used to grow plants in continuously aerated nutrient solution, with each tank holding up to 24 seedlings. The experiment included four replicates of each of the RILs, parental genotypes and check varieties in a completely randomised block design. *P. medicaginis* zoospore suspension culture was added at a final concentration in the nutrient solution of 1.5 × 10^5^ spores mL^−1^ for PRR disease development. The plants were assessed daily for wilting or plant death. The phenotyping experiment was terminated at 20 and 24 days for the RB and YG populations, respectively, based on the time of death of the PRR-susceptible RIL population parent. Stem canker length beginning from the hypocotyl region and proceeding upwards on the stem was measured for each plant using electronic digital 0–150 mm Vernier callipers.

### Genotyping data

Genotyping of all RIL progenies including the parental genotypes of YG and RB was performed by Diversity Arrays Technology Pty. Ltd. (Canberra, Australia) using chickpea DArT Seq version 1.0. Further details on the development of the linkage maps for both YG and RB RIL mapping populations and the molecular marker data are provided in Amalraj et al. [8].

### Statistical methods

#### Survival probability calculation

To ensure an effective quantitative measure was used for analyses of plant survival, Kaplan–Meier (KM) estimates of survival probability (KME-survival) [30] were calculated using the examined survival time of each of the plants. Let $t_{1} , \ldots , t_{n}$ be the time periods the experiment was examined over. The KM estimate of a plant surviving at time $t_{i}$ is then

$$S\left( {t_{i} } \right) = \mathop \prod \limits_{i = 1}^{n} \left( {1 - \frac{{d_{i} }}{{n_{i} }}} \right)$$

where $d_{i}$ are the number of plants that die during the $t_{i}$ th time period and $n_{i}$ are the number of plants at risk at the beginning of time period $t_{i}$. The ordered set of KM estimates $S(t_{1} ), \ldots , S\left( {t_{n} } \right)$ represent an estimate of the true survival function of the population when plants are infected with PRR. KM estimates approaching one signify early time of death and increased disease susceptibility, whereas estimates approaching zero suggest a prolonged survival time and resistance to PRR. This KM estimator also naturally allows censoring of individuals that did not germinate in the initial stages of the experiment through the appropriate reduction of the numbers of individuals at risk in the first time period. No additional transformation was required for this trait in the analysis models that follow.

#### Hydroponics univariate trait model

For each of the RIL populations, the KME-survival and canker length phenotypic traits were analysed using a linear mixed model (LMM) that partitioned and accounted for genetic and non-genetic sources of variation. Let $\varvec{y} = \left( {y_{1} , \ldots , y_{n} } \right)$ be the phenotypic response, then the linear mixed model was defined as

1 $$\begin{array}{*{20}c} {\varvec{y} = \varvec{X}\varvec{\tau} + \varvec{Z}\varvec{u} + \varvec{Z}_g \varvec{g} + \varvec{e}} \\ \end{array}$$

where $\varvec{X\tau }$ was the fixed component of the model and contained a population type factor to estimate the overall mean of the progeny population as well as means of the parental and control lines. The term $\varvec{Zu}$ was the random component containing factors to model sources of non-genetic variation including differences between the two sides of the controlled environment as well as differences between tanks containing the isolates used for inoculation. Additional extraneous variation was captured with the residual model error term, $\varvec{e}$, and was assumed to be distributed $\varvec{e }\sim N(\varvec{0},\sigma^{2} \varvec{I}_{n})$. Underlying genetic variation of the RIL population was modelled using the random component term $\varvec{Z}_g \varvec{g}$ where the genetic random effects, $\varvec{g}$, are an $r$ length vector and assumed to be distributed $\varvec{g }\sim N(\varvec{0},\sigma_{g}^{2} \varvec{I}_{r})$. The fixing of the parental and check varieties in the fixed component of the LMM ensured $\sigma_{g}^{2}$ reflects only the genetic diversity of the progeny population. Under this LMM structure, the effects, $\left( {\varvec{u},\varvec{ g},\varvec{ e}} \right),$ were considered to be mutually independent. For each population, best linear unbiased predictions (BLUPs) of the RIL progeny as well as their prediction error variances, were extracted from each of the fitted trait models and used to calculate broad sense generalized heritabilities with the formula derived in [10], namely

$$H_{g}^{2} = 1 - \frac{{PEV_{a} }}{{2\sigma_{g}^{2} }}$$

where $PEV_{a}$ is the average of the prediction error variances of all elementary contrasts between the progeny lines of the RIL population.

#### Hydroponics bivariate trait model

To understand the underlying genetic and phenotypic inter-relatedness between the KME-survival and canker length in each population, a bivariate LMM (BLMM) was fitted using an extension of (1). In this extension, the fixed component of the model consisted of an interaction of a two-level trait factor with the population type factor to ensure the means of the progeny, parental and check varieties for each of the traits were estimated separately for each trait. Extraneous sources of environmental variation were modelled for each trait using separate random effect terms. An important feature of the BLMM was the incorporation of terms that appropriately model the relatedness of the traits at the phenotypic and genetic level. Consequently, a multiplicative structure was assumed for the model error with a distribution $\varvec{e }\sim N(\varvec{0}, \varvec{R}_{b} \otimes \varvec{I}_{n})$, where $\varvec{R}_{b}$ is a 2 by 2 correlation matrix that reflects the residual variation within each trait as well as the residual phenotypic relationship between traits. Similarly, the genetic effects of the BLMM were assumed to have a multiplicative structure with distribution $\varvec{g }\sim N(\varvec{0}, \varvec{G}_{b} \otimes \varvec{ I}_{r})$, where $\varvec{G}_{b}$ is a 2 by 2 correlation matrix with diagonal elements reflecting the underlying genetic variation of each of the traits for the RIL population and an off-diagonal element capturing the true genetic correlation between the traits.

#### Quantitative trait loci analysis

For the phenotypic traits, KME-survival and canker length, a QTL analysis was conducted using the whole genome average interval mapping (WGAIM) approach of Verbyla et al. [31] and Verbyla et al. [32]. The WGAIM approach initially considers an extension of the LMM defined in (1) through a partitioning of the genetic effects, $\varvec{g}$, namely

2 $$\begin{array}{*{20}c} {\varvec{g} = \varvec{a} + \varvec{p}} \\ \end{array}$$

where $\varvec{a}$ is a set of additive genetic effects with assumed distribution $\varvec{a }\sim N(\varvec{0},\sigma_{a}^{2} \varvec{MM}^{T})$ and $\varvec{M}$ is a complete $(r \times q)$ matrix of interval markers (typically $q > r$) calculated using the rules defined in Verbyla et al. [31]. Here, $\varvec{MM}^{T}$ is an $(r \times r)$ additive relationship matrix used widely in the genetic association analysis literature [30, 31] to explore the underpinning genetic relationships between the lines and to provide computational efficiency to complex analyses. The remaining effects, $\varvec{p}$, on the right-hand side of (2) are polygenic or residual genetic effects and are assumed to be distributed $\varvec{p }\sim N(\varvec{0},\sigma_{p}^{2} \varvec{I}_{r})$. To determine whether interval markers were significantly linked to putative QTL, the additive variance parameter $\sigma_{a}^{2}$ was tested for significance by comparing the extended LMM with the baseline LMM defined in (1) through a simple likelihood ratio test. If significant, predicted interval marker effects are calculated through the back transformation $\tilde{\varvec{q}} = \varvec{M}^{T} \left( {\varvec{MM}^{T} } \right)^{ - 1} \varvec{\tilde{a} }$ [32–34], along with the predicted error variances, and outlier statistics are calculated for each interval marker using the methods derived in Verbyla et al. [31]. The interval marker with the largest outlier statistic is then removed from $\varvec{M}$ and placed as a separate random covariate in the extended LMM. This selection procedure was repeated until $\sigma_{a}^{2}$ was found to be non-significant. Selected interval markers are then independently tested using the techniques of Verbyla et al. [32] and summarized with their effect size, approximate contribution to the genetic variance and their LOD score.

#### Combined hydroponics and field trait model

An important component of this research is understanding the underlying genetic connection between the hydroponics phenotypic traits with the plant survival traits collected from multiple field environments and analyzed in Amalraj et al. [8]. Similar to the BLMM discussed earlier, the KME-survival and canker length can be combined with the plant survival field traits and analyzed using a multivariate multi-environment LMM (MVE-LMM). In this extension of (1) the fixed component of the MVE-LMM consisted of an interaction of a five-level trait factor with a population factor ensuring parental, control and progeny line means were estimated separately for each trait. The fixed component also contained terms to model extraneous environmental trends relevant for each of the traits. Other extraneous sources of variation associated with the field or glasshouse design as well as potential non-linear trends across row or columns of the layouts was modelled using separate random effects for each trait. Similar to the BLMM, terms were required for the MVE-LMM to ensure the genetic and phenotypic relatedness between traits was appropriately modelled. Specifically, if the phenotypic traits are ordered by field then glasshouse then the MVE-LMM residual error was assumed to be distributed

$$\varvec{e }\sim \varvec{ }N \left( {\left[ {\begin{array}{*{20}c} \varvec{0} \\ \varvec{0} \\ \end{array} } \right],\left[ {\begin{array}{*{20}c} { \oplus_{i = 1}^{3} \varvec{R}_{{f_i }} } & \varvec{0} \\ \varvec{0} & {\varvec{R}_{b} \varvec{ } \otimes \varvec{ I}_{n} } \\ \end{array} } \right]} \right)$$

where $\oplus_{i = 1}^{3} \varvec{R}_{f_i }$= diag $\left( \varvec{R}_{f_i } \right)$ and $\oplus$ is the so-called direct sum operator and used contextually in Butler et al. [27]. Here, $\varvec{R}_{f_i}$ is a residual correlation matrix containing for the $i$th field site containing a parameterization for an AR1 $\times$ AR1 (AR1 = autoregressive structure of order 1) to appropriately capture the correlation of the neighboring observations in the row and column directions of the field layout. The inclusion of the residual correlation structure $\varvec{R}_{b} \varvec{ } \otimes \varvec{ I}_{n}$ ensures the phenotypic relatedness of hydroponics traits are captured. The genetic effects of the MVE-LMM were assumed to have a multiplicative structure with distribution $\varvec{g }\sim N(\varvec{0},\varvec{G}_{m} \varvec{ } \otimes \varvec{ I}_{r})$ where $\varvec{G}_{m} \varvec{ }$ is a 5 $\times \varvec{ }$ 5 correlation matrix with diagonal elements consisting of genetic variances for each of the traits and off diagonal elements capturing the genetic correlation between traits. From the fitted MVE-LMM, the estimated genetic correlation matrix was extracted and summarized.

#### Computations

All univariate, bivariate and multivariate multi-environment linear mixed modelling was conducted using the ASReml-R package [35] available in the R Statistical Computing Environment (R Core Team 2018) [36]. ASReml-R uses a Residual Maximum Likelihood (REML) approach to estimation of model parameters [37] and is commercially available through VSN International (VSNi) at https://www.vsni.co.uk/software/asreml-r/. Univariate QTL analysis was conducted using the WGAIM R package [38] freely available from Comprehensive R Archive Network (CRAN) repository https://cran.r-project.org/web/packages/wgaim/index.html.

## Data Availability

The materials and datasets used and/or analyzed in this current study are available from the corresponding author on reasonable request.

## Abbreviations

PRR: Phytophthora root rot
DNA: deoxyribonucleic acid
KME-survival: Kaplan–Meier estimates of plant survival
QTL: quantitative trait loci
WGAIM: whole genome average interval mapping
KM: Kaplan–Meier
RIL: recombinant inbred line
MGB: minor groove binder
BLUPs: best linear unbiased predictions
BLMM: bivariate linear mixed model
LMM: linear mixed model
MVE-LMM: multi-environment linear mixed model
SCAR: Sequence Characterised Amplified Region
QDR: quantitative disease resistance
F_6_: Filial generation
PBA: Pulse Breeding Australia
DArT: Diversity Arrays Technology
LOD: Logarithm of the odds
REML: Residual Maximum Likelihood
